# Activities of Everyday Life with High Spinal Loads

**DOI:** 10.1371/journal.pone.0098510

**Published:** 2014-05-27

**Authors:** Antonius Rohlmann, David Pohl, Alwina Bender, Friedmar Graichen, Jörn Dymke, Hendrik Schmidt, Georg Bergmann

**Affiliations:** Julius Wolff Institute, Charitè – Universitätsmedizin Berlin, Berlin, Germany; The University of Queensland, Australia

## Abstract

Activities with high spinal loads should be avoided by patients with back problems. Awareness about these activities and knowledge of the associated loads are important for the proper design and pre-clinical testing of spinal implants. The loads on an instrumented vertebral body replacement have been telemetrically measured for approximately 1000 combinations of activities and parameters in 5 patients over a period up to 65 months postoperatively. A database containing, among others, extreme values for load components in more than 13,500 datasets was searched for 10 activities that cause the highest resultant force, bending moment, torsional moment, or shear force in an anatomical direction. The following activities caused high resultant forces: lifting a weight from the ground, forward elevation of straight arms with a weight in hands, moving a weight laterally in front of the body with hanging arms, changing the body position, staircase walking, tying shoes, and upper body flexion. All activities have in common that the center of mass of the upper body was moved anteriorly. Forces up to 1650 N were measured for these activities of daily life. However, there was a large intra- and inter-individual variation in the implant loads for the various activities depending on how exercises were performed. Measured shear forces were usually higher in the posterior direction than in the anterior direction. Activities with high resultant forces usually caused high values of other load components.

## Introduction

After spine surgery, patients should know which activities cause high spinal loads that may affect the clinical outcome. High spinal loads may lead to implant subsidence, pedicle screw loosening or even implant failure, and may also be a reason for low back pain. The maximum implant loads are also a prerequisite for the development and pre-clinical testing of spinal implants.

The spinal loads can be estimated using mathematical models [1]–[6]. However, it is difficult to account for all possible variations encountered during real exercises. Furthermore, only a limited number of activities, for which kinematics are well known, can be investigated.

*In vivo* intradiscal pressure measurements [7]–[10] can provide data on the overall load acting in a spine with non-degenerated discs. Many exercises in lying, sitting and standing positions were investigated using this method. In general, an exercise was measured once for each subject, and the number of subjects varied between 1 and 10 [8], [9], [11]. For example, the pressure value for standing was approximately 0.5 MPa [8], [10], and the pressure for forward bending was approximately 1.3 MPa [8]. Very high values were measured for different lifting exercises with a maximum pressure of 2.3 MPa when lifting a 19.8 kg case. For the indirect estimation of the spinal compressive force, using the intradiscal pressure and the cross-sectional area of the disc, a correction factor is needed [7], [12], [13]. This factor is subject-dependent and varies between 0.55 and 0.77 [7], [12], [14]; this results in an uncertainty of ±16.6% when the average value is chosen. The load taken over by the facet joints is usually unknown but affects the intradiscal pressure.

Spinal loads can also be measured by instrumented implants. The loads on internal spinal fixation devices [15], [16] and on a vertebral body replacement (VBR) [17]–[19] have been assessed in this way *in vivo.* The temporal course of all six load components was measured for different activities in multiple measuring sessions. It was observed that the load distribution between the instrumented implant and the spine depended on several factors, such as the surgical procedure, the remaining tissue, screw loosening or implant subsidence [17]. These factors are often unknown. The instrumented spinal fixation devices did not discriminate between activities with higher maximum spinal loads than during walking [20].

The aim of this study is to identify activities of everyday life that cause high load components, resultant bending moments and resultant forces on a VBR. This knowledge is required, e.g., to advise spine patients which activities to avoid, especially shortly after surgery.

## Materials and Methods

### Ethics Statement

The Ethics committee of Charité - Universitätsmedizin Berlin approved the implantation of the telemeterized implant in patients (Registry number 213–01/225–20). Prior to the surgery, the procedure was explained to the patients, and they gave their written consent for the implantation of the telemeterized VBR, participation in measurements and publication of their images.

### Instrumented implant and patients

A telemeterized VBR allowed the *in vivo* measurement of the 3 force and 3 moment components during activities of daily living. The implant was used in several studies [17]–[19], [21], [22] and was described in detail elsewhere [23]. Briefly, the telemeterized VBR was a modified SYNEX cage (Synthes Inc., Bettlach, Switzerland). Six strain gauges, a telemetry unit, and a coil for the inductive power supply were inserted in a hermetically sealed tube. The telemetry was active only within a magnetic field of 4 kHz. Screwed-on endplates of various heights enabled the intraoperative adaptation of the implant height to the defect length. Prior to implantation in patients, the VBR was calibrated by applying a large number of different known load combinations. The measuring sensitivities were better than 1 N and 0.01 Nm. The accuracy was approximately 2% for forces and 5% for moments relative to the calibration ranges of 3000 N and 20 Nm, respectively.

Five patients (WP1 – WP5) with A3-type compression fractures of a lumbar vertebral body (classification after Magerl et al. [24]) were treated with this implant. The vertebral body L1 was fractured in 4 patients and the vertebral body L3 in 1 patient (WP5). Further information about the patients, surgical procedure, and measurements are provided in Table 1. The fracture was first stabilized from the posterior, using an internal spinal fixation device. In a second surgery, parts of the fractured vertebral body and the adjacent intervertebral discs were removed, and the VBR was inserted in the prepared implant bed. Autologous bone material was then added to enhance the interbody fusion process.

**Table 1:** Data on patients, surgical procedures, number of measurements, load components, resultant force and resultant bending moment for lying relaxed in a supine position.

Parameter	Patient				
	WP1	WP2	WP3	WP4	WP5
Sex (M: Male, F: Female)	M	M	F	M	M
Age at the time of surgery (years)	62	71	69	63	66
Height (cm)	168	169	168	170	180
Body mass (kg)	66	74	64	60	63
Fractured vertebra	L1	L1	L1	L1	L3
Level of internal fixation device	T12–L2	T12–L2	T11–L3	T11–L3	L2–L4
Total no. of load measuring sessions	28	18	20	16	15
Number of trials	4219	2484	1802	2627	2454
Resultant force (N)	42	84	55	60	96
Bending moment (Nm)	0.22	0.86	0.43	0.23	0.55
Torsional moment (Nm)	−0.06	1.42	−0.43	0.22	−0.6
Shear force in ap-direction (N)	5	68	111	−39	−59
Lateral shear force (N)	−2	30	15	32	−49

### Measurements

Measurements were taken with an inductive power coil placed around each patient's trunk at the level of the VBR and a small loop antenna on each patient's back; both were fixed with a harness. The patients were videotaped during the measuring sessions, and the load-dependent signals from the telemetry were stored on the same videotape. The telemetry and the external equipment have been described in detail elsewhere [25]. Measurements were performed within a few days after surgery and up to 65 months postoperatively.

### Exercises studied

Implant loads were measured for daily activities in various body positions, such as standing, sitting and lying. The effects of lifting, carrying, and placing different weights with one or both hands were studied, and the effects of whole body vibrations and the wearing of a brace were also investigated. Other investigated exercises included level and staircase walking, physiotherapeutic exercises, and changing body positions. A total of approximately 1000 different combinations of activities and parameters were measured in 97 measuring sessions. The activities measured in a session were typically performed 2 or 3 times. Each of these trials was evaluated, and the maximum and minimum values of all 6 load components and the resultant forces and moments were stored in a database. Our database for the VBR comprises more than 13,500 datasets. A selection is available at www.orthoload.com.

#### Evaluation

The following load parameters were evaluated:

- maximum resultant force
- maximum resultant bending moment
- maximum torsional moment
- maximum shear force in the anterior direction
- maximum shear force in the posterior direction
- maximum lateral shear force

The resultant force is considered to be the most important load parameter of the VBR. For high values, it has nearly the same magnitude as the axial compression force. Therefore, this force component is not presented separately here. The resultant bending moment may have components in the frontal and the sagittal planes and can act around any horizontal axis. The bending moment is very important for designing a VBR and for the pre-clinical testing of the implant. However, it does not significantly affect the bending moment, which has to be transferred by the trunk. Thus, only data for the resultant bending moment are provided.

For each load component and for each patient, the average values of the single and resultant loads when lying relaxed in a supine position were measured to determine the preload value caused by the implantation of the VBR (Table 1). Then, the 10 exercises with the highest absolute differences to corresponding preload values were identified for each patient in the database.

Finally, the exercises with the highest values for a specific load parameter were pooled for 5 patients; the 10 exercises with the highest loads were then selected and are listed in a table together with the corresponding maximum values for each patient. However, a few special exercises, which were measured once in only 2 patients or a few times in only 1 patient, were not included in a top 10 list, even if a measured load component was one of the higher ones. For the resultant force, identified as the most significant load parameter in each exercise, the range of force measurements were also presented for each patient along with the corresponding number of measurements.

## Results

### Resultant force

**Figure 1:**
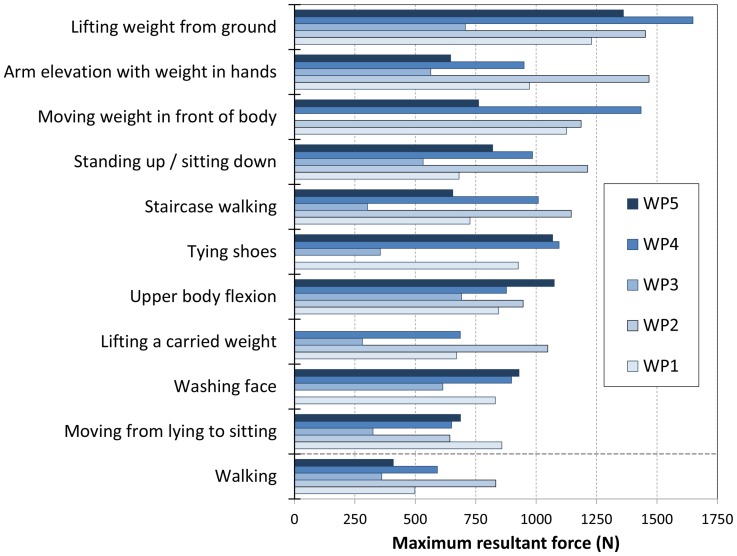
Ten activities with highest maximum resultant force. The maximum forces for the 5 patients are shown. For comparison, maximum forces for walking are given.

The highest resultant force (1650 N) was measured when lifting a crate weighing 10.8 kg from the ground (Table 2). In all 5 patients, the exercise of lifting a weight from the ground was 1 of the 10 exercises with the highest force (however, the maximum weight the patients were willing to lift varied between 4.3 and 10.8 kg). The other exercises with a maximum resultant force higher than 1200 N were as follows: the forwards elevation of straight arms with a weight up to 9.2 kg in hands, moving a weight up to 10.8 kg laterally in front of the body at the hip level, and changing the body position from sitting to standing. There were large inter-individual differences in the maximum resultant force (Figure 1). For all activities with a high resultant force, the magnitude was lowest in patient WP3. Walking, the most important daily activity, was ranked 11^th^. For comparisons, the belonging maximum resultant forces were also provided.

**Table 2:** Ten activities with the highest resultant implant force.

Top 10 activities with high resultant force	Number of patients with this activity in their top 10	Patient				
		WP1	WP2	WP3	WP4	WP5
Lifting weight from ground	5	1229–545 N	1452–1050 N	707–304 N	**1649**–1131 N	1361–732 N
		(7–10.8 kg)	(10–10.8 kg)	(4–4.3 kg)	(10 kg)	(4–10 kg)
		n = 60	n = 56	n = 17	n = 23	n = 72
Arm elevation forwards with weight in hands	5	972–611 N	**1467**–1135 N	564–216 N	950–680 N	646–323 N
		(9.2 kg)	(9.2 kg)	(3 kg)	(5 kg)	(5 kg)
		n = 26	n = 12	n = 13	n = 12	n = 17
Moving weight in front of the body	4	1126–758 N	1186–1141 N		**1434**–1288 N	762–726 N
		(10 kg)	(10.8 kg)	–	(Chair)	(10 kg)
		n = 6	n = 6		n = 4	n = 2
Standing up/sitting down	5	681–206 N	**1213**–586 N	532–113 N	985–344 N	820–277 N
		n = 61	n = 10	n = 50	n = 47	n = 24
Staircase walking	5	726–305 N	**1145**–624 N	302–81 N	1009–848 N	655–320 N
		n = 26	n = 19	n = 10	n = 6	n = 15
Tying shoes	4	926–585 N	–	355–251 N	**1095**–836 N	1068–652 N
		n = 4		n = 3	n = 5	n = 10
Upper body flexion	5	844–341 N	946–613 N	691–236 N	877–512 N	**1075**–318 N
		n = 88	n = 46	n = 31	n = 31	n = 28
Lifting a carried weight	4	690–361 N	**1048**–730 N	281–123 N	686–589 N	
		(9.2 kg)	(5–9.2 kg)	(0 kg)	(5 kg)	–
		n = 8	n = 9	n = 3	n = 2	
Washing face	4	831–712 N	–	614–507 N	898–764 N	**929**–579 N
		n = 5		n = 4	n = 7	n = 3
Moving from lying to sitting	4	**858**–170 N	643–460 N	325–130 N	650–361 N	687–192 N
		n = 16	n = 6	n = 14	n = 7	n = 6
Walking	2	*498–129 N*	**833**–348 N	361–44 N	*591–327 N*	*409–102 N*
		*n = 106*	n = 51	n = 75	*n = 75*	*n = 62*

Ranges of maximum forces, carried or lifted weight (in kg) and number (n) of measurements are given. For comparison, the data for walking are provided at the end.

*italic*: not in the top 10 of this patient; **bold**: peak values from all subjects for that activity.

### Resultant bending moment

The maximum resultant bending moment in the VBR (6.2 Nm) was measured for upper body flexion (Table 3). In all 5 patients, this exercise belonged to the top 10 activities with the highest bending moment. Other exercises with maximum bending moments higher than 5 Nm were as follows: the forwards elevation of straight arms with a weight in hands, lifting a weight from the ground, and putting a weight on a cupboard. All bending moments greater than 5 Nm were measured in patient WP2. For the other patients, the magnitudes of resultant bending moments for these exercises were much lower (Table 3).

**Table 3:** Ten activities with the highest resultant maximum bending moment in Nm.

Top 10 activities with high bending moment	Number of patients with this activity in their top 10	Patient				
		WP1	WP2	WP3	WP4	WP5
Upper body flexion	5	3.93	**6.23**	2.86	2.33	2.31
Arm elevation with weight in hands	4	3.23	**6.15**	1.63	1.61	*1.69*
		(9.2 kg)	(9.2 kg)	(1 kg)	(5 kg)	*(3 kg)*
Lifting weight from ground	5	4.92	**6.02**	1.44	4.85	3.96
		(10.8 kg)	(10.8 kg)	(4.3 kg)	(7 kg)	(4 kg)
Putting a weight on a cupboard	1	*1.54*	**5.72**	*−0.21*	*0.57*	*0.81*
		*(3 kg)*	(3 kg)	*(1 kg)*	*(3 kg)*	*(3 kg)*
Lateral bending	5	2.74	**4.08**	1.06	2.53	2.35
		(Standing)	(Standing)	(Sitting)	(Standing)	(Sitting)
Moving from lying to sitting	2	**4.06**	*4.0*	*0.57*	*1.42*	2.07
Tying shoes	3	**3.88**	*-*	*0.43*	2.56	3.10
Staircase walking	3	2.68	***3.88***	*0.73*	1.57	2.39
Moving weight in front of the body	3	**3.15**	*2.84*	*0.36*	3.11	2.20
		(10 kg)	*(Chair)*	*(3 kg)*	(Chair)	(10 kg)
Cleaning floor with mop	1	**2.86**	-	-	*0.73*	*1.3*

The carried weights (in kg) belong to the observed maximum moment.

*italic*: not in the top 10 of this patient; **bold**: peak values from all subjects for that activity

### Torsional moment

The highest torsional moment (3.5 Nm) was observed for the exercise ‘tying shoes’ (Table 4). Additional exercises with high torsional moments included the following: arm elevation with a weight in hands, moving from a lying to a sitting position, the axial rotation of the upper body and flexion of the upper body. Axial rotations of the upper body caused a maximum torsional moment of 3.15 Nm.

**Table 4:** Ten activities with the highest torsional moment in Nm.

Top 10 activities with high torsional moment	Number of patients with this activity in their top 10	Patient				
		WP1	WP2	WP3	WP4	WP5
Tying shoes	3	**3.51**	*-*	*0.29*	2.00	1.02
Arm elevation with weight in hands	5	**3.3**	2.83	0.59	1.33	1.02
		(5 kg)	(5 kg)	(3 kg)	(5 kg)	(3 kg)
Moving from lying to sitting	5	**3.16**	2.61	0.55	2.05	0.87
Axial rotation	3	*1.83*	**3.15**	0.61	1.64	*0.63*
Upper body flexion	3	**3.04**	*2.12*	0.55	*1.00*	0.78
Lateral bending	4	*1.49*	**2.89**	0.74	2.19	0.66
Staircase walking	3	*2.00*	**2.73**	*0.28*	1.83	0.82
Cleaning floor with mop	2	**2.55**	*-*	-	1.58	*0.6*
Lifting weight from ground	4	**2.77**	2.41	*0.42*	1.95	1.25
		(10 kg)	(7 kg)	*(4.3 kg)*	(10 kg)	(4 kg)
Walking	3	2.12	**2.38**	*0.29*	*1.23*	0.75

The carried weights (in kg) belong to the observed maximum moment.

*italic*: not in the top 10 of this patient; **bold**: peak values from all subjects for that activity

### Shear force in anterior direction

The highest anterior shear forces (130 N) were detected for ‘upper body flexion’ (Table 5). Lifting a weight from the ground and carrying a weight also caused maximum anterior shear forces greater than 100 N.

**Table 5:** Ten activities with the highest shear force (in N) in the anterior direction.

Top 10 activities with high anterior shear force	Number of patients with this activity in their top 10	Patient				
		WP1	WP2	WP3	WP4	WP5
Upper body flexion	5	88	**130**	52	81	36
Lifting weight from ground	4	90	122	50	**123**	*18*
		(10.8 kg)	(10.8 kg)	(4.3 kg)	(10.8 kg)	*(10.8 kg)*
Carrying weight in hands	1	*−11*	*−46*	*8*	**103**	*5*
		*(9.2 kg)*	*(9.2 kg)*	*(5 kg)*	(5 kg)	*(5 kg)*
Tying shoes	2	*−34*	-	*9*	**83**	28
Standing up/sitting down	5	**69**	45	51	62	34
Lateral bending	2	*3*	*−3*	*16*	**64**	32
		*(Sitting)*	*(Standing)*	*(Sitting)*	(Standing)	(Standing)
Axial rotation	3	*5*	30	*15*	**64**	28
		*(Sitting*)	(Sitting)	*(Sitting*)	(Standing)	(Sitting)
Staircase walking	3	35	*3*	*12*	**62**	33
Moving from lying to sitting	2	*13*	*5*	21	**56**	*19*
Washing face	1	*−10*	-	**52**	*16*	*22*

The carried weights (in kg) belong to the observed maximum force. Negative values indicate a shear force in the posterior direction.

*italic*: not in the top 10 of this patient; **bold**: peak values from all subjects for that activity

### Shear force in posterior direction

**Figure 2:**
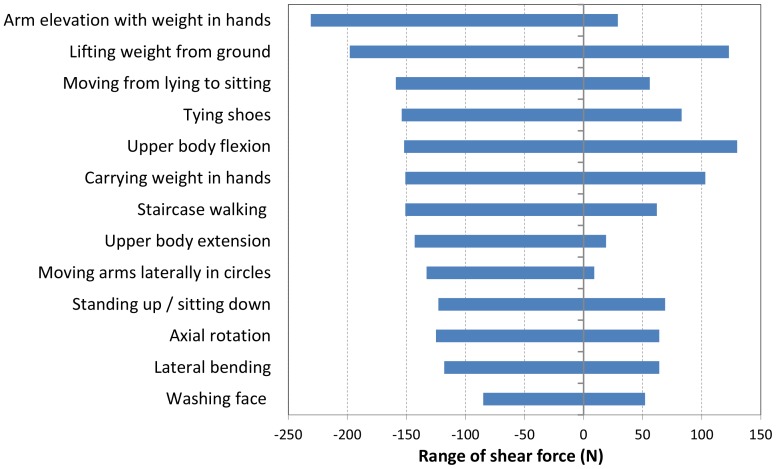
Maximum shear forces. Ranges of shear forces for the top 10 activities with maximum shear forces in the anterior (positive) and posterior (negative) directions. Seven activities were in the top 10 of maximum shear forces in both the anterior and posterior directions.

The maximum shear force in the posterior direction was much higher than that in the anterior direction (Figure 2). The highest posterior shear force (230 N) was found for the exercise ‘arm elevation with a weight in hands’ (Table 6). In all 5 patients, this exercise caused high shear forces. Other exercises with maximum posterior shear forces higher than 150 N included the following: lifting a weight from the ground, carrying a weight in a hand, moving from lying to sitting, tying shoes, upper body flexion and staircase walking.

**Table 6:** Ten activities with the highest shear force (in N) in the posterior direction.

Top 10 activities with high posterior shear force	Number of patients with this activity in their top 10	Patient				
		WP1	WP2	WP3	WP4	WP5
Arm elevation with weight in hands	5	−149	−**231**	−27	−93	−130
		(5 kg)	(9.2 kg)	(2 kg)	(5 kg)	(3 kg)
Lifting weight from ground	5	−164	−**198**	−17	−85	−172
		(10.8 kg)	(10.8 kg)	(4.3 kg)	(7 kg)	(4 kg)
Carrying weight in hands	2	−137	−**151**	*−4*	*−54*	*−10*
		(10.8 kg)	(10 kg)	*(10 kg)*	*(5 kg)*	*(5 kg)*
Moving from lying to sitting	2	−**159**	*−126*	−18	*−40*	*−2*
Tying shoes	3	−**154**	*-*	−14	*−51*	−59
Upper body flexion	4	−120	−**152**	−13	*−53*	−110
		(Standing)	(Standing)	(Standing)	*(Sitting)*	(Standing)
Staircase walking	3	−121	**−151**	−17	*−45*	*−11*
Upper body extension	3	*−84*	**−143**	−3	−57	*−31*
		*(Standing)*	(Sitting)	(Standing)	(Sitting)	*(Sitting)*
Moving arms laterally in circles	2	*−87*	**−133**	*4*	*−33*	−48
		*(Standing)*	(Standing)	*(Standing)*	*(Standing)*	(Sitting)
Standing up/sitting down	4	−113	***−123***	−8	−62	−77

The carried weights (in kg) belong to the observed maximum force.

italic: not in the top 10 of this patient; **bold**: peak values from all subjects for that activity

### Lateral shear force

The exercise with the highest shear force in the lateral direction (212 N) was ‘arm elevation with a weight in hands’ (Table 7). Additional exercises with lateral shear forces greater than 150 N were as follows: lifting a weight from the ground, moving a weight laterally in front of the body, and standing up or sitting down. All top 10 exercises of patient WP2 caused higher lateral shear forces than any exercise in other patients. The lateral bending of the upper body caused a maximum shear force of 122 N (rank 8).

**Table 7:** Ten activities with the highest lateral shear force in N.

Top 10 activities with high lateral shear force	Number of patients with this activity in their top 10	Patient				
		WP1	WP2	WP3	WP4	WP5
Arm elevation with weight in hands	5	87	**212**	43	76	61
		(5 kg)	(9.2 kg)	(3 kg)	(5 kg)	(3 kg)
Lifting weight from ground	5	84	**200**	53	102	69
		(10.8 kg)	(7 kg)	(4.3 kg)	(10.8 kg)	(10.8 kg)
Moving weight in front of the body	3	81	**175**	-	82	*48*
		(10 kg)	(10.8 kg)		(Chair)	*(10 kg)*
Standing up/sitting down	5	65	**163**	45	88	62
Carrying weight in hands	1	*43*	**113**	*28*	*66*	*35*
		*(9.2 kg)*	(10.8 kg)	*(5 kg)*	*(10.8 kg)*	*(10.8 kg)*
Staircase walking	4	63	**142**	40	95	*50*
Walking	3	*48*	**136**	36	*69*	63
Lateral bending	3	*50*	**122**	43	97	*41*
Axial rotation	3	*45*	**118**	*33*	92	55
		*(Standing)*	(Sitting)	*(Sitting)*	(Standing)	(Sitting)
Upper body flexion	4	65	***108***	57	104	81

The carried weights (in kg) belong to the observed maximum force.

*italic*: not in the top 10 of this patient; **bold**: peak values from all subjects for that activity

### Activities with high load components

Theoretically, up to 60 different exercises could be in the top 10 of the 4 load components, the resultant bending moment and the resultant force. However, 3 activities (i.e., lifting a weight from the ground, staircase walking and upper body flexion) were in all 6 top 10 lists (Table 8). Three activities (i.e., tying shoes, arm elevation with weight in hands and moving from lying to sitting) appeared 5 times, and 2 additional activities (standing up or sitting down and lateral bending) appeared 4 times in the top 10. Overall, 18 different activities had at least one load component in the top 10.

**Table 8:** All top 10 activities and the number of their occurrences in a top 10 list of the resultant force, resultant bending moment or one of the load components.

Activities in the top 10	Number of occurrences
Lifting weight from ground (up to 10.8 kg)	6
Staircase walking	6
Upper body flexion	6
Tying shoes	5
Arm elevation with weight in hands (up to 9.2 kg)	5
Moving from lying to sitting	5
Standing up/sitting down	4
Lateral bending	4
Carrying weight in hands (up to 10.8 kg)	3
Axial rotation	3
Moving weight in front of the body (up to 10.8 kg)	3
Washing face	2
Cleaning floor with mop	2
Walking	2
Lifting a carried weight (9.2 kg)	1
Putting a weight on a cupboard (up to 3 kg)	1
Upper body extension	1
Moving arms laterally in circles	1

## Discussion

The activities of daily living that caused high load components, high resultant bending moments and high resultant forces on a vertebral body replacement in the lumbar spine were selected from a database of more than 13,500 trials.

The 3 exercises with the highest resultant forces on the VBR were observed when lifting or carrying weights in front of the body. Similar exercises led to high intradiscal pressures [8]–[10]. In each of the 10 exercises that caused high axial compressive and high resultant forces, the center of mass of the upper body including the carried weight moved anteriorly. This is also true for the ‘changing body position’ [26] and ‘staircase walking’ [21] exercises. To stabilize the upper body, an anteriorly shifted center of mass required greater back muscle forces, which caused greater spinal compressive and resultant forces. Each of the overall top 10 activities with the highest resultant forces were also observed in at least 4 corresponding individual top 10 activities lists. However, the maximum force for the various exercises significantly varied intra- and inter-individually. On average, a top 10 activity was measured approximately 19 times per patient (for a range of 0–88 times). It should be noted that the spinal loads at the implant level were shared by the VBR, the internal fixation device, and the bone. Thus, the resultant force in the intact spine was always greater than the measured values.

The first 5 of the overall top 10 activities with the highest bending moments were measured in patient WP2. In that patient, the VBR was eccentrically implanted approximately 5 mm to the right. This offset was likely the cause for high bending moments. Seven of the top 10 activities with the highest bending moments were also in the top 10 for the highest resultant forces. For high loads, the resultant force nearly acts in the axial direction of the VBR. Therefore, a high bending moment in the VBR is not primarily caused by a changed inclination of the resultant force but by its lateral shift.

For the resultant force and the resultant bending moment, the exercises with the 10^th^ highest maximum value caused only approximately 50% or less of the maximum value measured for the exercise with the highest load. For the torsional moment, the corresponding value was approximately 69%. Surprisingly, for 2 patients, the ‘axial rotation of the upper body’ exercise did not cause a high torsional moment. Presumably, symmetrical exercises such as ‘arm elevation with a weight in hands’, ‘upper body flexion’ and ‘lifting a weight from the ground’ caused high torsional moments. This unexpected result may be due to such factors as slight deviations from the ideal axis of the VBR, an inclined implantation of the VBR, non-symmetrical muscle forces due to, e.g., the surgical approach, and a non-symmetrical performance of the exercise.

In accordance with our previous expectations, the greatest *anterior* shear forces were measured for the exercises ‘upper body flexion’ and ‘lifting a weight from the ground’. However, when the same exercises were performed on different days, *posterior* shear forces were sometimes higher. The direction of the shear force also varied for other exercises with high anterior shear forces. Often, the shear forces were already acting in the posterior direction before the exercise started. This indicates that muscle forces play an important role regarding the shear forces. The exercise ‘upper body flexion’ can be performed in different ways. The shape of the spine in the final position may slightly differ and affect the shear forces.

Surprisingly, for the top 10 activities, the magnitude of the shear force in the posterior was mostly higher than that in the anterior direction. The shear forces are also affected by the inclination of the VBR in the sagittal plane. For the 5 patients WP1 to WP5, this angle was approximately 10°, 5°, 0°, 2° and 1°, respectively. The highest posterior shear force value was found in patient WP2. In that patient, the VBR was eccentrically implanted, and the inclination angle in the sagittal plane was 5°. In patient WP5 and in patients WP1, WP2, WP3, and WP4, the VBR was implanted at levels L3 and L1, respectively. At level L1, the spine is slightly curved; in a standing position, this vertebra is mostly posteriorly inclined, which may be the reason for high shear forces in the posterior direction for exercises causing a high resultant force. In this case, a part of that resultant force acts in the posterior direction.

Surprisingly, the exercise ‘lateral bending of the upper body’ was only ranked 8^th^ among the exercises with the highest lateral shear force. A few symmetrical exercises involving additional weights led to higher shear forces. For each of the top 10 exercises, the highest lateral shear force was measured in patient WP2 (Table 7). In that patient, the VBR was eccentrically implanted; this is likely the cause for the observed high bending moment and high lateral shear forces. Axial rotation is expected to cause high lateral shear forces when the gap in the facet joint is closed. However, this activity was only ranked 9^th^. For healthy intervertebral disc levels, the lateral shear force may be higher because the posterior implants likely reduced the motion in the patients.

Activities associated with high shear forces in anterior direction may promote the development of degenerative spondylolisthesis, while activities with high lateral shear forces and synchronous high torsional moments may promote the development of degenerative scoliosis. However, further studies are required to substantiate these hypotheses.

In patient WP5, the L3 vertebral body was replaced, while the L1 vertebral body was replaced in the other patients. In an upright body position, the L3 is usually more horizontal than the L1. This may be why shear forces in the anterior and lateral directions and the torsional moment were mostly smallest in patient WP5.

Due to osteoporosis, the internal fixators in patient WP4 were mounted 2 levels above and 2 levels below the fractured vertebra L1. These adjustments may account for the high *anterior* shear forces and low shear forces in the *posterior* direction.

For many activities, the bending and torsional moments in patient WP1 were high. In that patient, the inclination of the VBR in the sagittal plane was highest (approximately 10°). X-ray images suggested that the VBR was slightly eccentrically implanted. Because of this implantation, the compressive force also causes a bending and torsional moment. The kyphosis angle of the thoracic spine increased during the measuring period [17]. This led to increased resultant forces for standing and walking in the temporal course of the measurements.

For the top 10 activities, the lowest resultant forces were always measured in patient WP3. This patient was not able to carry the same maximum weights as the other patients. In most exercises, the load components for patient WP3 were also lower than that for the other patients.

Elderly people often develop a slight scoliosis in the lumbar region. This would increase the shear forces and the torsional moment.

For activities involving a carried weight, the maximum value of a load component did not correspond to exercises using the heaviest weight. In fact, the way an exercise was performed often had a stronger influence than the magnitude of the carried weight.

There were some limitations to this study. Implant loads were only measured for a small sample size of 5 patients. These patients were older than 60 years at the time of surgery. The surgical procedure was not exactly the same in all patients, e.g., in 4 patients, the vertebral body L1 was replaced, while in one patient, the vertebral body L3 was replaced. The location of the internal fixator was also variable due to osteoporosis, e.g., twice mounted two levels above and two levels below the fractured vertebra. Not all patients were able to perform all exercises. The number of repetitions of an activity varied widely. The postoperative time of measurements also varied from a few days to 65 months. The fraction of the total spinal load, taken over by the VBR, was likely not constant over the wide postoperative time range [17]. Furthermore, although not all activities of daily living could be measured, we tried to investigate as many as possible (approximately 1000 different activity and parameter combinations). Due to the necessary external equipment, only activities that were performable in a gym hall were measured.

In summary, high forces on a VBR were measured for activities of daily life. The activities with the highest resultant force have in common that the center of mass of the upper body (and a carried weight) was shifted anteriorly. High resultant forces were usually accompanied by high values of the single load components. Thus, only 18 activities were present in the 6 top 10 lists. There were large intra- and inter-individual variations in the implant loads. Finally, it should be noted that the VBR measured only the implant and not the complete spinal load.
